# Circadian distribution of carbohydrate intake during adolescence and association with risk markers of type 2 diabetes in adulthood—the role of chronotype

**DOI:** 10.1007/s00394-026-04056-x

**Published:** 2026-08-03

**Authors:** Nicole Jankovic, Sarah Schmitting, Ines Perrar, Eva Hohoff, Azadeh Lesani, Janina Goletzke, Bianca Stutz, Christian Herder, Stefan A. Wudy, Ute Nöthlings, Ute Alexy

**Affiliations:** 1 Institute of Nutritional and Food Sciences-Nutritional Epidemiology, (DONALD Study), University of Bonn, Heinstück 11, 44225 Dortmund, Germany; 2 Faculty of Natural Sciences, Institute of Nutrition, Consumption and Health, Paderborn University, Warburger Strasse 100, 33098 Paderborn, Germany; 3 Faculty of Health, Institute for Integrative Health Care and Health Promotion, Witten/Herdecke University, Alfred-Herrhausen-Straße 50, 58448 Witten, Germany; 4 Institute for Clinical Diabetology, German Diabetes Center, Leibniz Center for Diabetes Research at Heinrich Heine University Düsseldorf, Auf’m Hennekamp 65, 40225 Düsseldorf, Germany; 5 German Center for Diabetes Research (DZD), Partner Düsseldorf, Ingolstädter Landstraße 1, 85764 Neuherberg, Germany; 6 Department of Endocrinology and Diabetology, Medical Faculty and University Hospital Düsseldorf, Heinrich Heine University Düsseldorf, Moorenstraße 5, 40225 Düsseldorf, Germany; 7 Pediatric Endocrinology and Diabetology, Laboratory for Translational Hormone Analytics, Peptide Hormone Research Unit, Center of Child and Adolescent Medicine, Justus Liebig University Giessen, Feulgenstraße 12, 35392 Giessen, Germany; 8 Institute of Nutritional and Food Sciences-Nutritional Epidemiology, University of Bonn, Friedrich-Hirzebruch-Allee 7, 53115 Bonn, Germany

**Keywords:** Carbohydrates, Glycemic index, Adolescents, Chronotype, Type 2 diabetes risk

## Abstract

**Background:**

Adolescence is a critical phase characterized by temporary insulin resistance and a tendency for a later chronotype, potentially influencing the risk of developing type 2 diabetes (T2D) later in life. This study explores how morning and evening carbohydrate (CHO) intake during adolescence are prospectively related to T2D risk factors—such as insulin resistance (HOMA2-IR), liver fat indices, and subclinical inflammation— in adulthood while considering the role of chronotype.

**Methods:**

This analysis utilizes data from 224 DONALD study participants (58% female) who provided 3-day weighed dietary records during adolescence (median age = 12 years) and a blood sample in adulthood (median age = 22 years). Owing to detected interactions between the timing of CHO intake i.e. CHO_evening(E%)_–CHO_morning(E%),_ (eveningness in CHO intake) and chronotype, analyses were stratified accordingly.

**Results:**

An inverse relationship was found between morning CHO intake (before 11 a.m.) and adult HOMA2-IR levels (beta and 95% CI for high (T3) vs. low (T1): −0.12 (−0.21; −0.04), p_*trend*_ = 0.01). For individuals with an earlier chronotype, a higher "eveningness in CHO intake" was linked to higher HOMA2-IR (p_*trend*_ = 0.03). Conversely, those with a later chronotype exhibited the lowest HOMA2-IR in the median "eveningness in CHO intake" category (T2). No significant associations were observed between evening CHO intake and other risk markers.

**Conclusion:**

Consuming CHOs in the morning during adolescence is associated with lower HOMA2-IR in adulthood, irrespective of chronotype. However, the favorable morning CHO intake amount appears to vary by chronotype, indicating a personalized approach may be beneficial.

**Supplementary Information:**

The online version contains supplementary material available at 10.1007/s00394-026-04056-x.

## Introduction

Insulin sensitivity exhibits a diurnal rhythm and decreases over the day [1, 2]. In line with this, Kessler et al. [3] showed an increase in insulin resistance if most carbohydrates (CHO) were consumed in the evening compared to morning intake. Regarding the prevention of Type 2 Diabetes (T2D) also CHO quality, i.e. dietary glycemic index (GI) [4, 5] or glycemic load (GL) are discussed. Current evidence, albeit preliminary, suggests that health benefits regarding T2D risk may particularly arise from the avoidance of large amounts of higher-GI-CHO (GI ≥ 55) consumed in the evening. This finding was reported earlier for the prospective association between CHO exposure during adolescence and T2D risk markers in adulthood [6] and in an adult population of six shift workers within a randomized controlled trial [7]. Hence, the circadian intake of CHO and its quality may play an important role in the prevention of T2D. Focusing on adolescents is of tremendous importance as this age may constitute a vulnerable time window for the development of chronic diseases e.g. T2D [8]. Reasons may relate to a transient insulin resistance [9], a delayed mid-point of sleep (i.e. late chronotype), which in turn may increase the risk of overweight [10] and unfavorable body compositional developments during adolescence [11]. The chronotype describes individual differences in sleep timing (earlier or later), and is determined by genetics, age, sex, and environment [12]. Chronotype is primarily governed by the internal biological clock (circadian rhythm), located in the suprachiasmatic nucleus (SCN) of the hypothalamus, which is strongly influenced by light exposure. Sunlight acts as the most important external cue (or “Zeitgeber”) for aligning the internal clock with the 24-h day [13]. But also, the timing of food intake acts as a Zeitgeber and could potentially influence the balance between the internal and external clock. Later chronotypes have an increased T2D risk [14], which may be explained via unfavorable body compositional developments [15] and glycemic responses [16]. In a recent randomized controlled cross-over trial, we found that university students with early and late chronotypes exhibit different 2-h glucose responses to a high glycemic index meal, depending on whether the meal was consumed in the morning or evening [16]. Hence, the influence of CHO intake or quality on T2D risk may depend on timing of food intake and chronotype as hypothesized earlier [6, 16]. To the best of our knowledge the association between diurnal CHO intake during adolescence and adult T2D risk under consideration of chronotype remains unstudied. Given the elevated risk profile of adolescents for developing T2D, we aimed to investigate the long-term association between adolescent meal timing focusing on CHO intake (quantity and quality) as an early-life exposure that may influence later metabolic risk through long-term circadian and metabolic programming. Homeostasis model assessment 2—insulin resistance (HOMA2-IR), hepatic steatosis index (HIS), fatty liver index (FLI) [17] and biomarkers of subclinical inflammation are established T2D risk factors in adulthood [6]. We hypothesized that the associations between diurnal CHO intake (quantity and quality) and diabetes risk factors differ by chronotype, meaning adolescents with an earlier chronotype may benefit most by consuming CHO earlier in the day (before 11 a.m.) while adolescents with a later chronotype may not. We further hypothesized that CHO quality, specifically glycemic index (GI), also modifies these associations, such that higher-GI CHO intake may be particularly important for adolescents with earlier chronotypes than for those with later chronotypes.

## Methods

### DONALD study

The DONALD study is an ongoing dynamic (open) cohort study in which information on nutrition, growth, development and metabolism of healthy children and adolescents in Dortmund (Germany) is collected. The study was initiated in 1985 as a cross-sectional sample of children and adolescents. Ever since 35–40 infants were newly recruited each year. Eligibility criteria are healthy infants with willing parents to participate in a long-term study and at least one parent with sufficient knowledge of the German language. Invitations for examinations during adolescence take place annually including 3-day weighed dietary records, anthropometric measurements, 24-h urine samples, lifestyle interviews and medical examinations. Parental examinations take place every 4 years. The study is non-invasive during childhood and adolescence. Since 2005, participants aged ≥ 18 years have been invited for subsequent examinations including fasting blood samples collections. Further details of the study have been described elsewhere [18, 19]. The DONALD study is registered as a cohort study in the German Register of Clinical Trials (DRKS-ID: DRKS00029092).

### Study population

At the time of data preparation for this analysis (July 2019) the DONALD study sample consisted of N = 2,376 participants. Of these, blood samples for additional analyses of T2D risk markers were available for N = 484 participants, who had already reached adulthood (Suppl. Fig. S1). Participants were eligible for the present analysis if they filled out.

1. At least one Munich Chronotype Questionnaire (MCTQ) from age 9 years (girls) or 10 years (boys) onwards up to adulthood (maximum age 48 years), and
2. At least one 3-day weighed dietary record collected during adolescence (girls: 9–15 years; boys: 10–16 years).

This resulted in a sample of 242 participants. Per participant, between one and four MCTQs and between one and seven dietary records were available. Repeated measures of MCTQs and dietary records were averaged at the individual level. After exclusion of participants with incomplete or invalid MCTQ data [20] (n = 4) and MCTQs collected within two weeks after daylight saving time changes (n = 11), the final analytical sample comprised 227 participants for analyses of steatotic liver indices and inflammatory biomarkers.

The sample size for insulin resistance outcomes was slightly smaller due to fasting glucose values below the calculation threshold [6] or insufficient blood volume. The final sample for insulin resistance analyses consisted of 224 participants.

### Dietary assessment during adolescence

Dietary intake was assessed by 3‐day weighed dietary records on three consecutive days, including information on the timing of meal consumption. At the time of the annual examination at the study center, participants are asked to record their dietary intake. Participants are free to choose the days of recording for each visit. Participants were instructed by dietitians to weigh all consumed foods and beverages, including leftovers, to the nearest 1 g. For this purpose, they received regularly calibrated electronic food scales (initially Soehnle Digita 8000 (Leifheit AG, Nassau, Germany), now WEDO digi 2000 (Werner Dorsch GmbH, Muenster/Dieburg, Germany). Semiquantitative measures (e.g., number of spoons, cups) were allowed when exact weighing was not possible. In addition, information on recipes and on the types and brands of food items consumed was requested. The dietary records were collected as well as reviewed by the dietitians and analyzed using the continuously updated inhouse nutrient database “LEBensmittelTABelle” (food table, LEBTAB [19, 21]). It includes information from standard nutrient tables, product labels, or recipe simulations based on the listed ingredients and nutrients.

### Exposure variables: timing and quality of CHO intake during adolescence

#### Definition of morning and evening intake

Morning intake was defined as food consumption between the age-specific end of the night and 11:00 a.m., while evening intake was defined as consumption between 6:00 p.m. and the age-specific start of the night. Age-specific night periods were derived from average timing of first and last eating occasions in the DONALD cohort and were defined in accordance with previous studies [22, 23].

#### CHO quantity and quality

For each CHO-containing food consumed in the defined morning or evening time windows, published glycemic index (GI) values were assigned [24, 25]. using standardized procedures [26]. Glycemic load (GL) was calculated by multiplying the CHO content (g) by the GI of each food. Dietary GI for each time window was calculated as total GL divided by total CHO intake of the corresponding day-time window.

CHO intake was further classified into low-GI (GI < 55) [25] and moderate- to high-GI (GI ≥ 55) sources. Accordingly, morning and evening intake of low-GI CHOs and higher-GI CHOs (g/day) were calculated.

“Eveningness in CHO intake” was defined as the difference between the percentage of total daily CHO intake consumed in the evening and in the morning. An analogous approach was applied to CHO quality “eveningness in CHO quality” (Diederichs et al. 2018). Participants were categorized into tertiles, with negative values indicating morningness and positive values indicating eveningness of CHO intake.

### Blood analyses during adulthood

Venous blood samples were drawn from the age of ≥ 18 years after an overnight fast. Blood samples were centrifuged at 4 °C and frozen at -80 °C in the DONALD Study Center. Fasting plasma glucose levels were determined using a Roche/Hitachi Cobas c 311 analyser (Basel, Switzerland). Plasma insulin concentrations were measured at the Laboratory for Translational Hormone Analytics of the University of Giessen using an immunoradiometric assay (IRMA, DRG Diagnostics, Marburg, Germany). All other measurements were performed at the German Diabetes Center with the following assay characteristics [27–30]: plasma activities of alanine‐aminotransferase (ALT), aspartate‐aminotransferase (AST), gamma‐glutamyltransferase GGT), plasma triglycerides (TG) and plasma high‐sensitivity C‐reactive protein (hsCRP) with the Roche/Hitachi Cobas c311 analyser (Roche diagnostics, Mannheim, Germany), plasma high-sensitivity IL-6 using the Human IL-6 Quantikine HS ELISA, plasma adiponectin with the Human Total Adiponectin/Acrp30 Quantikine ELISA, serum leptin with the Leptin Quantikine ELISA (all from R&D Systems, Wiesbaden, Germany) and serum IL-18 using the Human IL-18 ELISA kit from MBL (Nagoya, Japan).

### Definition of outcome variables: T2D risk markers during adulthood

Four T2D risk markers were derived: HOMA2-IR, HIS, FLI, PIS. Each of the risk markers indicate a higher risk for developing T2D with increasing levels. Insulin resistance was assessed using the updated HOMA2-IR calculator (University of Oxford) [31] based on fasting insulin and blood glucose [32]. Indices for steatotic liver and subclinical inflammation were calculated as follows [6]:

HSI: 8 × ALT / AST + BMI.

(+ 2, if female; + 2 if T2D [33], the latter was not applicable to the DONALD sample, since there were no participants with T2D).

FLI: e^x^ / (1 + e^x^) × 100.

With x = 0.953 × ln(TG) + 0.139 × BMI + 0.718 × ln(GGT) + 0.053 × waist circumference—15.745 [6, 34]

Pro-inflammatory score (PIS): (z-hsCRP + z-IL-6 + z-IL18 + z-adiponectin × (− 1) + z-leptin) / 5 [6, 35].

For the calculation of the outcome variables anthropometric data during adulthood were incorporated. The assessment methods equal those during childhood and adolescence and will be elaborated below. During adulthood BMI (kg/m^2^) was calculated.

### Chronotype variables during adolescence and adulthood (potential effect modifier/covariate)

The MCTQ [12, 36] includes questions on sleep and wake times separately for weekdays and weekend. The individual chronotype (continuously in h:min) was calculated as the midpoint of sleep, i.e., the half-way point between sleep-onset and sleep-end on free days (MSF) [36]. The MSF is corrected for “oversleep” on free days, for people who sleep longer on school-free days than on schooldays, to account for sleep-debt accumulated over the week (MSFsc) [12]. Chronotype is also shaped by social factors, for instance school schedules that may conflict with an individual's internal timing. This mismatch between the internal and external clock can lead to “social jetlag” (SJL) which, in the current manuscript, was defined as the absolute difference (initial number of participants with negative SJL n = 4) between the midpoint of sleep on free days and that on schooldays [37] and may be an important covariate.

Adolescent boys and girls differ in their individual level of lateness; girls tend to be earlier in comparison to boys [38]. To increase the final analytic sample from *n* = 143 to n = 224 and 227, respectively, and to ensure adequate statistical power, all available MCTQ assessments from adolescence through adulthood were included. To account for age- and sex-related differences in chronotype, MSFsc residuals (adjusted for age and sex) were derived for each observation and then averaged across repeated measures at the individual level. Then the sample was classified in participants with an earlier or later chronotype by median split [15]. We assessed the reliability of the averaged chronotype variable by an overall and categorized correlation matrix between the averaged chronotype and the chronotype that accompanied the dietary data during adolescence. Finally, we re-analyzed the significant findings of the current manuscript in an adolescent-only sensitivity analyses, using only adolescent chronotype assessments, despite the resulting smaller sample size.

### Assessment of potential confounders (adolescence)

Anthropometric measurements were performed by trained nurses according to standard procedures, with the participants dressed in underwear only, and barefoot. Standing height was measured to the nearest 0.1 cm (digital stadiometer: Harpenden Ltd., Crymych, UK) and body weight to the nearest 0.1 kg (electronic scale: model 753 E; Seca, Hamburg, Germany). Waist circumference was measured at the midpoint between the lower rib and the iliac crest to the nearest 0.1 cm. Measurements of skinfold thicknesses were taken on the right side of the body at the biceps, triceps, subscapular and suprailiac sites to the nearest 0.1 mm (Holtain caliper: Holtain Ltd., Crymych, UK). From these measures, adolescent BMI (kg/m^2^) and its sex‐ and age‐specific SD ‐ scores (SDS) were calculated using current German BMI standards [39]. All anthropometric measures obtained during adolescence that coincided with dietary assessments were averaged at the individual level.

On a child’s admission to the study, information on gestational characteristics and birth anthropometrics (e.g. birth weight) were derived from a standardized document (Mutterpass), given to all pregnant women in Germany. Moreover, parents were interviewed concerning the child’s early life data as well as family and socio‐economic characteristics (e.g. maternal education or employment, smoking in the household) at regular intervals. Individual total kcal intake and other dietary variables during adolescence e.g. animal protein intake were calculated from weighed dietary records as individual means of three record days [20]. Physical activity during adolescence and adulthood was assessed using an interview-based questionnaire at the study center, which inquired about participation in organized and non-organized sports [18]. Energy expenditure was estimated by multiplying the mean duration of activity (hours/day) by the estimated basal metabolic rate (using age and sex depended algorithms according to Schofield [40], kcal/hour) and metabolic equivalents (MET, according to Ainsworth [41]. When multiple measures were available during adolescence, information was averaged and considered as a covariate in the model.

### Statistical analysis

All statistical analyses were performed using SAS® procedures (version 9.4; Cary, NC, USA). Tests for interaction were performed by the inclusion of an interaction term between the exposure variables and sex or chronotype. We additionally stratified the data by chronotype to confirm the statistical result [42]. The significance level was set at *p* < 0.05.

There was no indication for interaction by sex or chronotype regarding the association between morning or evening CHO intake or quality and T2D risk factors. However, we observed significant interaction between chronotype and “eveningness in CHO intake”. Therefore, we present all associations besides “eveningness in CHO intake” and HOMA2-IR on the overall sample while the stratified results are attached (Suppl. Table S1).

We applied multivariable regression models to assess the prospective associations between adolescent dietary intake in the morning and evening (i.e., CHO, GI, GL, Low-GI-CHO or higher-GI-CHO) and adulthood risk markers for T2D. Transformations in the outcome variables (square root (HOMA2-IR), reciprocal transformation (HSI), double-log (FLI) and log transformation (PIS)) were performed prior to the analyses for achieving normal distribution in favor of meeting the assumptions for regression analyses. Furthermore, values of IL-6 (N = 4) and adiponectin (N = 1) were winsorized, i.e. outliers by the sex-specifically closest value fitting a normal distribution, which is in accordance with previous analyses performed in DONALD [6]. Energy adjustment was performed on all dietary exposure variables (except GI and E%) using the residual method [43]. All GI variables represent age (years), and sex standardized (mean = 0, SD = 1) values that were averaged over the time of adolescence, where applicable. Covariates for adjustment were selected according to known predictors of CHO intake and T2D risk factors [6, 28, 44]. All of the selected variables were included in a directed acyclic graph (DAG) to identify a minimally sufficient adjustment set (MSAS) considering all known relationships among the identified variables [45]. The DAG (Suppl. Fig. S2) revealed three possible adjustment sets that we tested all, revealing comparable results, so that we finally decided to use the following MSAS including: BMI-SDS at baseline (i.e. during adolescence), age at blood withdrawl (range 18–46) and date of dietary record (improved precision of the estimate), birth weight, parental employment (yes/No), parental education (years of education), metabolic equivalents (MET-min), residuals of animal protein, sex, smoking in the household (yes/no), social jetlag, total kcal intake [20].

To assess the relative importance of morning and evening intake we incorporated both time windows simultaneously in the analyses by examining the associations between “eveningness in CHO intake” and T2D risk factors. Only the association between “eveningness in CHO intake” and HOMA2-IR qualified for a chronotype stratified analyses by a significant p-value on the one hand and opposite effect measures on the other hand.

In separate sensitivity analyses we excluded those participants with underreported records which were defined as an inappropriate TEI in relation to the estimated basal metabolic rate [40]. Pediatric cut-offs from Sichert-Hellert et al. [46] were applied to identify underreporter. Adapted cut-offs for over-reporting [22, 46] did not reveal any over-reported records. For further sensitivity analysis, we excluded those participants who reported parental diabetes and conducted analyses of biomarkers of subclinical inflammation using cross-sectional dietary data from adulthood. Finally, we calculated baseline characteristics of included and excluded participants.

## Results

Baseline characteristics of the adolescent sample are presented according to chronotype in Table 1. Most participants (67%) provided more than one 3-day dietary records during adolescence (median = 7, min = 1 and max = 7). Physical activity was higher in adolescents with a later chronotype as compared to adolescents with an earlier chronotype. Furthermore, adolescents with a later chronotype had larger intakes in CHO and lower intakes of fat during the morning resulting in greater “morningness in CHO intake” as presented by negative values for the “eveningness in CHO” score. Follow-up characteristics for adulthood are shown in Table 2. During this period, physical activity was higher in earlier chronotypes in comparison to later chronotypes. Differences in metabolic risk variables between chronotypes were small. However, adult HOMA2-IR levels were higher in later chronotypes.

**Table 1:** Baseline characteristics of N = 227 adolescence of the DONALD study according to chronotype^a^

	Chronotype	
	Earlier (113)	Later (114)
*General characteristics*		
Females	65 (58)	66 (58)
Age adolescence (years)^b^	12 (12; 13)	12 (12; 13)
*Sleep characteristics (hh:mm)* ^a^		
MSFsc	3:21 (2:54; 3:41)	04:46 (04:13; 05:26)
Social Jetlag	1:15 (00:47; 1:44)	2:00 (01:27; 02:40)
*Early life factor*		
Birth weight (kg)	3.39 (3.11; 3.73)	3.42 (3.09; 3.75)
*Family characteristics and lifestyle factors*		
Maternal education, ≥ 12 years of schooling	60 (53)	74 (65)
Maternal employment (yes)	69 (61)	88 (77)
Smoking in the household (yes)*	28 (31)	20 (23)
Parental diabetes	1 (0)	0 (0)
MET (min) ^b^	842.4 (528.8; 1350.0)	978.5 (705.8; 1347.2)
*Body composition during adolescence* ^b^		
BMI (kg/m^2^)	18.4 (16.9; 20.2)	18.8 (17.0; 20.6)
BMI-SDS (kg/m^2^)	0.08 (−0.51; 0.68)	0.24 (−0.52; 0.75)
*Nutrition parameters* ^a^		
Daily E intake (kcal)	1843 (1627; 2109)	1912 (1675; 2089)
CHO (E%)	50 (48; 54)	52 (49; 53)
Total protein (E%)	13 (12; 14)	13 (12; 14)
Total animal protein (E%)	8 (7; 9)	8 (7; 9)
Total fat (E%)	35 (32; 38)	35 (32; 37)
E before 11 a. m. (kcal)	522 (454; 640)	488 (402; 581)
E before 11 a. m. (E%)	28 (25; 31)	27 (23; 30)
GI	55.8 (53.6; 57.9)	55.9 (53.6; 59.0)
GL (g)	38.9 (33.8; 47.3)	38.2 (30.5; 46.4)
CHO low-GI (E%)^c^	26 (20; 35)	26 (18; 34)
CHO higher-GI (E%)^c^	42 (35; 50)	38 (30; 46)
CHO (E%)	53 (49; 57)	55 (51; 59)
Protein (E%)	12 (11; 13)	12 (11; 14)
Animal protein (E%)	7 (6; 8)	7 (5; 8)
Fat (E%)	33 (29; 37)	31 (27; 34)
E after 6 p. m. (kcal)	547 (446; 647)	568 (487; 672)
E after 6 p. m. E%	29 (24; 32)	31 (27; 34)
GI	56.5 (54.7; 58.7)	56.1 (54.2; 58.1)
GL (g)	37.8 (30.1; 45.9)	37.3 (30.2; 46.5)
CHO low-GI (E%)^c^	17 (15; 21)	19 (15; 23)
CHO high-GI (E%)^c^	31 (28; 35)	28 (24; 33)
CHO (E%)	49 (45; 53)	48 (44; 52)
Protein (E%)	14 (13; 15)	14 (13; 16)
Animal protein (E%)	9 (7; 10)	9 (7; 10)
Fat (E%)	36 (32; 39)	37 (33; 40)
Eveningness in CHO intake (g/d)	−7 (−18; 12)	1 (−11; 15)
Eveningness in CHO intake (%)	−4 (−9; −0)	−7 (−13; −2)
*Diet assessment quality*		
Underreporting	7 (6)	4 (4)

Characteristics are presented as mean ± std, median (Q1, Q3) or n and (%)

DOrtmund Nutritional and Anthropometric Longitudinally Designed (DONALD);

Midpoint of sleep correct for oversleep during the weekend (MSFsc); metabolic equivalent of task (MET); Body Mass Index (BMI); Standard Deviation Score (SDS); Energy (E); Carbohydrates (CHO); Saturated Fatty Acids (SFA); Glycemic Index (GI); Glycemic Load (GL)

^a^Chronotype was assessed by the Munich Chronotype Questionnaire and defined as age and sex adjusted midpoint of sleep on free days corrected for sleep debt on workdays (MSFsc) using all measurements from adolescence up to adulthood applying regression analyses. Earlier and later chronotypes based on the averaged median values of MSFsc

^b^Mean over six years (♀: 9–15 years, ♂: 10–16 years)

^c^Distinction between CHO intake from low‐ and higher‐GI food sources with a GI of 55 as cut‐off

^d^Content of a product is estimated by summing up the CHOs stemming from energetic sweeteners, for example, sugar, honey or syrup, according to the definition in Cummings & Stephen (Cummings, JH & Stephen, AM (2007) Carbohydrate terminology and classification. Eur J Clin Nutr 61, Suppl. 1, S5–S18.)

**Table 2:** Sample characteristics for adulthood (N = 227) of the DONALD Study according to chronotype^a^

	Chronotype	
N	Earlier (113)	Later (114)
*General characteristics*		
Age at blood withdrawal (years)	23 (18; 30)	21 (18; 24)
Follow-up (years)	11 (7; 18)	8 (6; 12)
*Lifestyle factors during adulthood*		
No alcohol intake ^b^	14 (16)	32 (31)
Excessive alcohol consumption (yes)^c^	5 (6)	9 (9)
Currently smoking (yes)	19 (17)	23 (21)
MET-minutes	743 (348; 1314)	681 (441; 1175)
*Body composition during adulthood*		
BMI (kg/m^2^)	22.9 (21.2; 25.4)	22.9 (21.2; 24.9)
*Weight status during adulthood* ^d^		
Underweight < 18.5 (kg/m^2^)	7 (6)	4 (4)
Normal weight 18.5–24.9 (kg/m^2^)	75 (66)	82 (72)
Overweight WHO 25.0–30.0 (kg/m^2^)	25 (22)	21 (18)
Obesity > 30 (kg/m^2^)	6 (5)	7 (6)
*Risk markers of type 2 diabetes*		
HOMA2-IR	1.35 (1.07; 1.67)	1.48 (1.20; 1.82)
HSI	32.4 (29.2; 35.4)	31.6 (29.0; 34.8)
FLI	7.6 (4.0; 20.7)	8.3 (4.6; 18.3)
Pro-inflammatory score	−0.09 (−0.35; 0.26)	−0.07 (−0.40; 0.30)
*Single risk markers*		
Glucose (mg/dl)	90 (86; 97)	91 (86; 96)
Insulin (µlU/ml)	10.4 (8.2; 12.9)	11.5 (9.2; 14.1)
Adiponectin (µg/l)	7.3 (5.4; 10.4)	6.6 (4.7; 9.2)
Triglycerides (mg/dl)	92 (62; 114)	85 (63; 129)
AST (Units/L)	22 (18; 26)	22 (18; 26)
ALT (Units/L)	20 (16; 26)	19 (16; 27)
GGT (Units/L)	14 (11; 20)	13 (11; 19)
hsCRP (mg/l)	1.3 (0.4; 2,4)	0.8 (0.5; 2.5)
Il-6 (pg/ml)	0.7 (0.4; 1.0)	0.7 (0.5; 1.1)
IL-18 (pg/ml)	256 (193; 330)	237 (197; 294)
Leptin (µg/l)	8.6 (3.6; 17.8)	10.3 (4.0; 16.6)

Characteristics are presented as median (Q1, Q3) or n and (%)

DOrtmund Nutritional and Anthropometric Longitudinally Designed (DONALD); MET, metabolic equivalent of task; BMI, body mass index; WHO, world health organisation; HOMA, homeostatic model assessment; IR, insulin resistance; HSI, hepatic steatosis index; FLI, fatty liver index; AST, aspartate-aminotransferase; ALT, alanine-aminotransferase; GGT, γ-glutamyltransferase; CRP, C-reactive protein; IL, interleukin

^a^Chronotype was assessed by the Munich Chronotype Questionnaire and defined as age and sex adjusted midpoint of sleep on free days corrected for sleep debt on workdays (MSFsc) using all measurements from adolescence up to adulthood applying regression analyses. Earlier and later chronotypes based on the averaged median values of MSFsc;^b^ > 18 years; ^c^for women > 10 g/d and > 20 g/d for men; ^d^according to WHO definition

Regression analyses for morning or evening intakes of CHO and CHO quality did not show any significant interaction by chronotype as shown in Table 3. Results of the stratified analyses for earlier and later chronotypes mainly pointed in the same direction, especially for the morning intakes of CHO and CHO quality. Also, in the stratified analyses by chronotype only CHO intake in the morning showed a significant inverse association with HOMA2-IR (Suppl. Table. S1). Hence, we present the results unstratified for chronotype. Morning CHO intake during adolescence was inversely associated with adult HOMA2-IR levels (P_trend_ = 0.005). In addition, also adolescent GL in the morning was inversely associated with HOMA2-IR (P_trend_ = 0.004). There were no significant associations regarding the amount or quality of CHO intake in the morning or evening and HSI, FLI or inflammation as elaborated further below.

**Table 3:** Regression coefficients and 95% confidence intervals between residual morning (before 11 a.m.) or evening (after 6 p.m.) carbohydrate (CHO) intake or quality during adolescence and HOMA2-IR in adulthood under consideration of chronotype (N = 224), the DONALD study

	Low exposure (T1)	Medium exposure (T2)	High exposure (T3)	*p for Trend* ^*1*^	p interaction chronotype^2^
MORNING					
*CHO*					0.67
*Median CHO (E%)*	49 (46; 51)	54 (53; 56)	60 (58; 62)		
crude^3^	Ref	−0.00 (−0.08; 0.07)	−0.04 (−0.12; −0.03)		
Model 1^4^	Ref	−0.05 (−0.13; 0.03)	−0.12 (−0.21; −0.04)	*0.005*	
HOMA2-IR (ls means)	2.96 (2.41; 3.55)	2.57 (2.12; 3.07)	1.95 (1.54; 2.41)		
*Glycemic Index (GI)*					0.88
*Median GI*	*52 (51; 54)*	*56 (55; 57)*	*60 (58; 61)*		
crude^3^	Ref	−0.03 (−0.11; 0.05)	−0.02 (−0.09; 0.06)		
Model 1^4^	Ref	−0.12 (−0.34; 0.09)	−0.08 (−0.30; 0.13)	*0.63*	
HOMA2-IR (ls means)	2.69 (2.22; 3.19)	2.28 (1.85;2.75)	2.43 (1.99; 2,92)		
*Glycemic Load (GL)*					0.94
*Median GL*	*34 (28; 41)*	*36 (30 / 43)*	*44 (39 / 53)*		
crude^3^	Ref	−0.02 (−0.10; 0.06)	−0.05 (−0.12; −0.03)		
Model 1^4^	Ref	−0.06 (−0.13; 0.02)	−0.12 (−0.20; −0.03)	*0.004*	
HOMA2-IR (ls means)	2.99 (2.46; 3.57)	2.50 (2.06; 3.00)	1.97 (1.56; 2.44)		
*CHO with low-GI*					0.65
*Median low-GI-CHO (E%)*	*14 (11; 17)*	*21 (19; 23)*	*29 (26; 33)*		
crude^3^	Ref	−0.06 (−0.13; 0.02)	−0.05 (−0.12; −0.03)		
Model 1^4^	Ref	−0.03 (−0.11; 0.04)	−0.03 (−0.11; −0.05)	*0.24*	
HOMA2-IR (ls means)	2.62 (2.16; 3.13)	2.36 (1.93; 2.84)	2.41 (1.97; 2.89)		
*CHO with higher-GI*					0.88
*Median high-GI-CHO (E%)*	*25 (22; 28)*	*33 (31; 34)*	*39 (36; 43)*		
crude^3^	Ref	−0.03 (−0.10; 0.05)	0.01 (−0.07; 0.08)		
Model 1^4^	Ref	−0.02 (−0.09; 0.05)	−0.04 (−0.11; 0.03)	*0.26*	
HOMA2-IR (ls means)	2.64 (2.17; 3.15)	2.49 (2.05; 2.98)	2.26 (1.83; 2.74)		
EVENING					
*CHO*					0.12
*Median CHO(E%)*	*42 (40; 45)*	*48 (47; 50)*	*54 (52; 57)*		
crude^3^	Ref	−0.04 (−0.11; 0.04)	0.00 (−0.07; 0.08)		
Model 1^4^	Ref	−0.02 (−0.10; 0.06)	0.01 (−0.07; 0.08)	*0.87*	
HOMA2-IR (ls means)	1.52 (1.39; 1.65)	1.48 (1.36; 1.61)	1.53 (1.41;1.67)		
*Glycemic Index (GI)*					0.66
*Median GI*	*53 (52; 54)*	*56 (56; 57)*	*59 (58; 61)*		
crude^3^	Ref	−0.04 (−0.12; 0.03)	−0.00 (−0.08; 0.08)		
Model 1^4^	Ref	−0.05 (−0.13; 0.03)	0.01 (−0.07; 0.09)	*0.81*	
HOMA2-IR (ls means)	1.54 (1.41; 1.68)	1.42 (1.30; 1.55)	1.57 (1.44;1.70)		
*Glycemic Load (GL)*					0.39
*Median GL*	*32 (27; 40)*	*36 (30; 42)*	*46 (37; 54)*		
crude^3^	Ref	−0.04 (−0.14; 0.01)	−0.03 (−0.10; 0.05)		
Model 1^4^	Ref	−0.04 (−0.12; 0.03)	−0.03 (−0.10; 0.05)	*0.50*	
HOMA2-IR (ls means)	1.56 (1.43; 1.70)	1.47 (1.35; 1.60)	1.50 (1.38; 1.63)		
*CHO with low-GI*					0.10
*Median low-GI-CHO (E%)*	*13 (10; 15)*	*18 (17; 21)*	*23 (22; 26)*		
crude^3^	Ref	−0.06 (−0.14; 0.02)	−0.04 (−0.12; 0.03)		
Model 1^4^	Ref	−0.06 (−0.14; 0.01)	−0.04 (−0.12; 0.03)	*0.25*	
HOMA2-IR (ls means)	1.60 (1.47; 1.73)	1.45 (1.33; 1.57)	1.49 (1.37; 1.61)		
*CHO with higher-GI*					0.90
*Median high-GI-CHO (E%)*	*23 (21; 26)*	*30 (29; 32)*	*36 (33; 38)*		
crude^3^	Ref	−0.03 (−0.12; 0.04)	−0.03 (−0.11; 0.05)		
Model 1^4^	Ref	−0.03 (−0.11; 0.04)	−0.02 (−0.10; 0.05)	*0.49*	
HOMA2-IR (ls means)	1.56 (1.43; 1.70)	1.47(1.35; 1.60)	1.50 (1.37; 1.63)		

DOrtmund Nutritional and Anthropometric Longitudinally Designed (DONALD); T, Tertile; HOMA, homeostatic model assessment; IR, insulin resistance; MET, metabolic equivalent of task; BMI, body mass index

Median values of intake refer to median and (Q1; Q3) in each tertile of the respective exposure. ^1^ p for trend are based on linear regression models using the median of the categorical exposure variables. ^2^ interactions were tested in the crude model ^3^crude model adjusted for age at blood withdrawl and sex. ^4^ Crude model additionally adjusted for BMI-SDS, residuals of animal protein, total kcal intake, smoking (yes/no), MET-min, parental employment, education, birthweight, social jetlag, date of dietary record

**Fig. 1:**
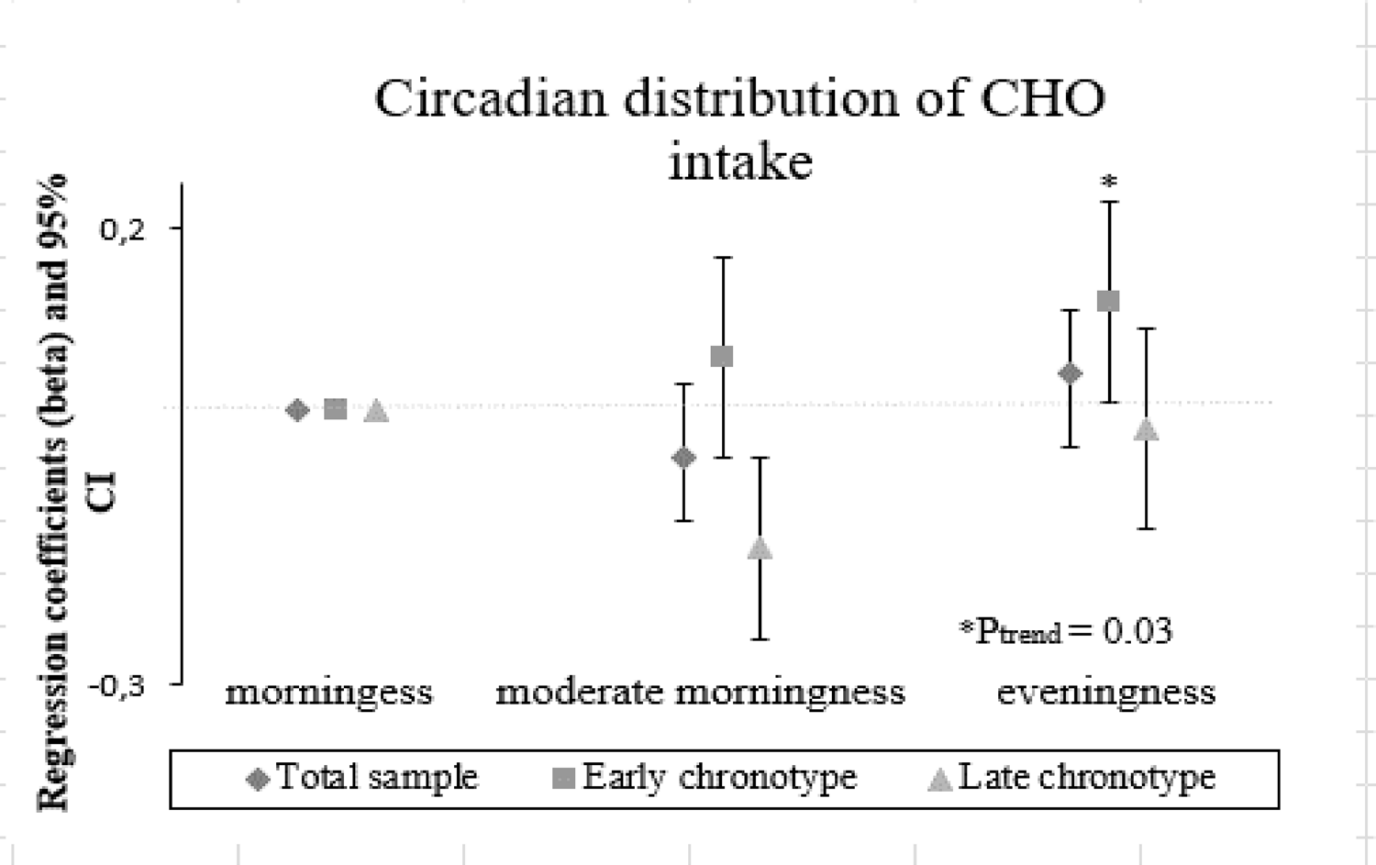
Regression coefficients and 95% CI of the associations between circadian intake of carbohydrate (CHO) as measured by "Eveningness in CHO intake"* and HOMA2-IR stratified by chronotype. *The circadian distribution of diurnal CHO intake was measured by 'eveningness in CHO intake' (E%) was calculated as: CHO_evening_–CHO_morning_. Overall values were: T1 = −13.04 (−15.41; −10.38), T2 = −6.22 (−7.66; −3.85), T3 = 2. 38 (−0.81; 4.94). Classification into tertiles showed negative values for T1 and T2 indicating either “morningness in intake” or “moderate morningness in intake” while T3 is represented by positive values indicating “eveningness in intake”, indeed. Values are presented as beta estimates and 95% confidence intervals across tertiles obtained from linear regression. P for trend were derived by using the median of the categorical exposure variables in the model. Models were adjusted for sex, age at blood withdrawl, SDS-BMI, residual of animal protein intake, total kcal/d, smoking (yes/no), MET-min, parental employment, parental education, birth weight. P_interaction_ for the association between 'eveningness in CHO' and HOMA2-IR with chronotype < 0.001 and *P_trend_ = 0.03

In Fig. 1, we considered morning and evening CHO intake simultaneously in terms of “eveningness in CHO intake” for which we observed a significant interaction by chronotype (p_interaction_ < 0.001). While persons with earlier chronotypes showed higher HOMA2-IR levels with increasing lateness in CHO intake (p_trend_ = 0.03), participants with later chronotypes showed a significant inverse association between moderate “morningness in CHO intake” with HOMA2-IR levels represented by a u-shaped association. There was no significant interaction by chronotype regarding “eveningness in CHO quality” and HOMA2-IR (Suppl. Table S2). Non-significant associations between “eveningness in CHO intake” and PIS, HIS or FLI are shown in Suppl. Table S3 to S5.

The conclusions of the sensitivity analyses remained robust. Results of the reliability study regarding the averaged chronotype are summarized hereafter. The correlations between the first and last MSFsc measurement was r = 0.59 and correct classification into adolescents with an earlier or later chronotype considering one measurement during adolescence against the averaged value across all other available measurements was 85%. The adolescent-only sensitivity analyses showed associations that were directionally consistent with the primary analyses. For the “eveningness in CHO intake”, the test for trend was no longer significant among earlier chronotypes. In contrast, among later chronotypes, the association between higher CHO intakes towards evening hours remained directionally consistent and approached a significant linear trend.

## Discussion

No previous study on the association between CHO quantity or quality and T2D risk evaluated the role of chronotype, yet. Hence, the results presented in the current manuscript are of particular interest. The current study shows that CHO intake in the morning during adolescence is associated with lower HOMA2-IR values in adulthood irrespective of chronotype. However, taking the relative importance of morning and evening CHO intake into account, as measured by “eveningness in CHO intake”, revealed differences by chronotype in the association with HOMA2-IR. For participants with an earlier chronotype, later consumption of CHO towards evening hours (after 6 p.m.) relative to morning hours (before 11 a.m.) showed an association with higher HOMA2-IR levels. For participants with a later chronotype an u-shaped association was observed, which was represented by a significant inverse association in the medium category of “eveningness in CHO intake” and HOMA2-IR. Noteworthy, we found neither any associations between the amount of CHO intake or CHO quality in the evening and adult HOMA2-IR nor an association with Indices for steatotic liver or subclinical inflammation.

The consumption of CHO during morning hours appears to benefit the reduction of T2D risk factors in healthy populations [47–49]. An impact of high CHO intake in the morning hours on HOMA2-IR is biologically plausible considering the circadian pattern of glucose metabolism since insulin sensitivity is higher during morning hours [1, 2]. In line with this, also glucose tolerance follows a circadian rhythm with a peak during the day and a drop towards sleeping hours [50]. This is largely attributable to a lower pancreatic ß-cell function in the evening resulting in a lower early-phase insulin response [51]. Of note, the consumption of higher amounts of CHO with a high GI in the morning hours showed an inverse association with HOMA2-IR, which was underlined by the significant inverse association observed between GL and HOMA2-IR. These findings were unexpected given the existing evidence linking carbohydrate (CHO) quality to an increased risk of type 2 diabetes (T2D) [5, 52, 53]. However, they may be explained by the greater short-term satiety associated with high–glycemic index (GI) intake, particularly when consumed in the morning [54–56]. A protein pre-load prior CHO consumption for instance may positively influence the insulin response and lead to improved postprandial glycemia [57]. The combination of cereals or bread as the primary CHO source together with dairy products as the main protein source represents a traditional German breakfast pattern [58] and may partly account for our observed associations with glycemic index (GI) and glycemic load (GL).

In contrast to the present analysis, Diederichs et al. did not consider chronotype in their study. They examined a slightly different sample of the DONALD study and observed that CHO low in GI consumed in the evening were beneficial for HSI while CHO with higher GI consumed in the evening were associated with higher HSI levels. In the current study, we found no associations for HSI, FLI or biomarkers of subclinical inflammation. One reason may be related to the uncertainty in causality between metabolic dysfunction- associated steatotic liver disease (MASLD previously NAFLD) and diabetes [59]. The difference in results with Diederichs et al. could also be attributable to (i) the selection of the sub-sample as we took the availability of chronotype data into account or (ii) the lack in repeated measures in order to account for habitual GI and GL intake which may particularly be of importance for T2D risk as shown earlier [6, 60].

In contrast to our initial hypothesis, we could not identify interaction by chronotype for the morning or evening exposure in CHO intake, but there was an interaction by chronotype for the association between “eveningness in CHO intake” and HOMA2-IR. “Eveningess in CHO intake” allows for a cautious interpretation regarding the diurnal intake of CHO. Hence, while participants with earlier chronotype appear to benefit the most from a prominent morning CHO intake instead of a later CHO intake, participants with a later chronotype appear to have more benefit from a moderate morning CHO intake in relation to a later intake. Moderate “morningness in CHO intake” would allow for example a later first breakfast instead of an early breakfast at home with their parents and an additional breakfast at school. German schools provide a breakfast break but no free breakfast. Free school breakfast may be a beneficial policy measure to support the reduction of T2D risk. More evidence regarding breakfast and early markers for T2D risk is necessary [61]. Adolescents with a later chronotype tend to skip an early breakfast [20]. Hence, the outlined scenario we suggested above would probably match with the biological clock of participants with later chronotypes. The results of the stratified analyses are in line with previous studies. Xiao et al. [48] presented a clear benefit of increased morning energy and CHO intake (Q5 vs Q1) regarding overweight and obesity for early chronotypes, while adolescents with a later chronotype appeared to have most benefits of a later morning intake (Q5 vs Q1). Accordingly, we also showed in one previous study the benefit of later energy intake in line with the underlying biological clock of adolescents with later chronotypes for the association with FFMI [15]. Concerning the exposure of interest of the current study, a recently published cross-over study confirmed a better glucose response for earlier chronotypes consuming a higher GI test meal in the morning (7 a.m.) but not in the evening (8 p.m.), while adolescents with a later chronotype showed an increased glucose response at both daytimes [16], indicating that participants with a later chronotype may be particularly sensitive for a detrimental glucose response consuming a high GI meal very early in the morning and also late in the day. The presented results are difficult to compare due to differences in study design. Nevertheless, both studies could show differences in chronotype regarding preferable eating times and glucose metabolism.

Some strengths and limitations of the present analysis should be discussed. A major strength of the present analysis is the longitudinal cohort design of the DONALD study that allows prospective analyses. Furthermore, for most of the participants the study offers detailed day-time specific nutritional data of participants who repeatedly assess their dietary intake which incorporates a training effect and may potentially lead to less bias. Besides using a validated method for dietary assessment [62] also chronotype is assessed by use of a validated questionnaire [63, 64]. Finally, all blood samples were analyzed in the same laboratories and the same personal, reducing the risk for inter and intra-variability in analytes. There are also some limitations attached to our study: As with every other observational study we cannot be sure of a causal relationship since not all the requirements according to the Bradford Hill criteria are met [65]. Also, reverse causation cannot entirely be excluded but it is unlikely given the large body of evidence regarding nutrition and its preventive characteristics for chronic non-communicable diseases. Data of the exposure was gathered during a time when our participants often skip study measures since other life aspects are more appealing during adolescence. This, in combination with the assessment of chronotype from age 9 years onwards, reduced the sample by more than 50%. However, considering the baseline characteristics of the sample, we could not observe large differences between excluded and included participants. The generalizability of results may be limited due to our young and healthy sample with mostly European ancestry characterized by an above-average socioeconomic status, which may also account for the non-significant association especially between CHO intake and subclinical inflammation due to a low amount of heterogeneity in the outcome.

We operationalized chronotype in the same manner as in our previous publication [15] by applying a median split to ensure adequate sample sizes in each group. For future analyses using larger datasets, it may be preferable to assess chronotype using more than two categories, and the development of empirically derived cut-offs to define earlier and later chronotypes would be beneficial. One potential concern is that defining chronotype by averaging all available measurements may obscure temporal variability. However, given the moderate correlation between individual chronotype assessments and the averaged, age- and sex-adjusted chronotype measure, we consider this approach sufficiently reliable for distinguishing between participants with earlier versus later chronotypes. Moreover, heritability estimates for chronotype are approximately 50% [66], suggesting that an averaged measure may potentially capture the underlying genetically driven chronotype. In addition to the increased statistical power afforded by this summary measure, we cannot exclude the possibility of nondifferential misclassification bias [4], which may have attenuated the observed associations, as suggested by the adolescent-only sensitivity analyses for “eveningness in CHO intake” among later chronotypes. Future longitudinal studies require repeated measurements of both chronotype and meal timing and preferable more than one measure on T2D risk markers to increase variability in the data.

## Conclusion

Overall, we found an inverse association between CHOs consumed in the morning hours during adolescence and adult insulin resistance. Earlier chronotypes showed a linear trend in association with HOMA2-IR in favor of morning CHO consumption while later chronotypes would benefit from a smaller amount in CHO consumption during the morning hours. A lower CHO intake in the morning hours for later chronotype may be realized by the consumption of a later first breakfast at school instead of an early first breakfast at home.

## Supplementary Information

Below is the link to the electronic supplementary material.

Supplementary file1 (DOCX 443 KB)

## Data Availability

Data of the DONALD study is available upon request to: epi@uni-bonn.de.

## References

[1] Saad A, Dalla Man C, Nandy DK et al (2012) Diurnal pattern to insulin secretion and insulin action in healthy individuals. Diabetes 61:2691–2700. 10.2337/db11-1478 22751690 10.2337/db11-1478 PMC3478548

[2] Sonnier T, Rood J, Gimble JM et al (2014) Glycemic control is impaired in the evening in prediabetes through multiple diurnal rhythms. J Diabetes Complicat 28:836–843. 10.1016/j.jdiacomp.2014.04.001 10.1016/j.jdiacomp.2014.04.001 24835190

[3] Kessler K, Hornemann S, Petzke KJ et al (2017) The effect of diurnal distribution of carbohydrates and fat on glycaemic control in humans: a randomized controlled trial. Sci Rep 7:44170. 10.1038/srep44170 28272464 10.1038/srep44170 PMC5341154

[4] Sluijs I, Beulens JWJ, van der Schouw YT et al (2013) Dietary glycemic index, glycemic load, and digestible carbohydrate intake are not associated with risk of type 2 diabetes in eight European countries. J Nutr 143:93–99. 10.3945/jn.112.165605 23190759 10.3945/jn.112.165605

[5] Jenkins DJA, Willett WC, Yusuf S et al (2024) Association of glycaemic index and glycaemic load with type 2 diabetes, cardiovascular disease, cancer, and all-cause mortality: a meta-analysis of mega cohorts of more than 100 000 participants. Lancet Diabetes Endocrinol 12:107–118. 10.1016/S2213-8587(23)00344-3 38272606 10.1016/S2213-8587(23)00344-3

[6] Diederichs T, Herder C, Roßbach S et al (2017) Carbohydrates from sources with a higher glycemic index during adolescence: is evening rather than morning intake relevant for risk markers of type 2 diabetes in young adulthood? Nutrients. 10.3390/nu9060591 28604592 10.3390/nu9060591 PMC5490570

[7] Morgan LM, Shi J-W, Hampton SM et al (2012) Effect of meal timing and glycaemic index on glucose control and insulin secretion in healthy volunteers. Br J Nutr 108:1286–1291. 10.1017/S0007114511006507 22176632 10.1017/S0007114511006507

[8] Buyken AE (2015) Kohlenhydratqualität und Krankheitsentstehung. Kinder-und Jugendmedizin 15(01):15–21. 10.1055/s-0038-1629249

[9] Goran MI, Gower BA (2001) Longitudinal study on pubertal insulin resistance. Diabetes 50:2444–2450. 10.2337/diabetes.50.11.2444 11679420 10.2337/diabetes.50.11.2444

[10] Siervogel RM, Demerath EW, Schubert C et al (2003) Puberty and body composition. Horm Res Paediatr 60(suppl 1):36–45. 10.1159/000071224 10.1159/000071224 12955016

[11] Jankovic N, Schmitting S, Krüger B et al (2022) Changes in chronotype and social jetlag during adolescence and their association with concurrent changes in BMI-SDS and body composition, in the DONALD Study. Eur J Clin Nutr 76:765–771. 10.1038/s41430-021-01024-y 34702962 10.1038/s41430-021-01024-y PMC9090626

[12] Roenneberg T, Kuehnle T, Juda M et al (2007) Epidemiology of the human circadian clock. Sleep Med Rev 11:429–438. 10.1016/j.smrv.2007.07.005 17936039 10.1016/j.smrv.2007.07.005

[13] Roenneberg T, Merrow M (2016) The circadian clock and human health. Curr Biol 26:R432–R443. 10.1016/j.cub.2016.04.011 27218855 10.1016/j.cub.2016.04.011

[14] Yu JH, Yun C-H, Ahn JH et al (2015) Evening chronotype is associated with metabolic disorders and body composition in middle-aged adults. J Clin Endocrinol Metab 100:1494–1502. 10.1210/jc.2014-3754 25831477 10.1210/jc.2014-3754

[15] Jankovic N, Schmitting S, Stutz B et al (2024) Alignment between timing of “highest caloric intake” and chronotype in relation to body composition during adolescence: the DONALD Study. Eur J Nutr 63:253–265. 10.1007/s00394-023-03259-w 37863858 10.1007/s00394-023-03259-w PMC10799146

[16] Stutz B, Krueger B, Goletzke J et al (2024) Glycemic response to meals with a high glycemic index differs between morning and evening: a randomized cross-over controlled trial among students with early or late chronotype. Eur J Nutr. 10.1007/s00394-024-03372-4 38605233 10.1007/s00394-024-03372-4 PMC11329680

[17] Busca C, Sánchez-Conde M, Rico M et al (2022) Assessment of noninvasive markers of steatosis and liver fibrosis in human immunodeficiency virus-monoinfected patients on stable antiretroviral regimens. Open Forum Infect Dis 9:ofac279. 10.1093/ofid/ofac279 35873289 10.1093/ofid/ofac279 PMC9297309

[18] Kroke A, Manz F, Kersting M et al (2004) The DONALD study. Eur J Nutr 43:45–54. 10.1007/s00394-004-0445-7 14991269 10.1007/s00394-004-0445-7

[19] Perrar I, Alexy U, Nöthlings U (2023) Cohort profile update-overview of over 35 years of research in the dortmund nutritional and anthropometric longitudinally designed (DONALD) study. Eur J Nutr. 10.1007/s00394-023-03290-x 38151532 10.1007/s00394-023-03290-x PMC10948456

[20] Roßbach S, Diederichs T, Nöthlings U et al (2018) Relevance of chronotype for eating patterns in adolescents. Chronobiol Int 35:336–347. 10.1080/07420528.2017.1406493 29231764 10.1080/07420528.2017.1406493

[21] Sichert-Hellert W, Kersting M, Chahda C et al (2007) German food composition database for dietary evaluations in children and adolescents. J Food Compos Anal 20:63–70. 10.1016/j.jfca.2006.05.004

[22] Roßbach S, Diederichs T, Bolzenius K et al (2017) Age and time trends in eating frequency and duration of nightly fasting of German children and adolescents. Eur J Nutr 56:2507–2517. 10.1007/s00394-016-1286-x 27492443 10.1007/s00394-016-1286-x

[23] Diederichs T, Perrar I, Roßbach S et al (2018) In adolescence a higher ‘eveningness in energy intake’ is associated with higher total daily energy intake. Appetite 128:159–166. 10.1016/j.appet.2018.05.142 29842968 10.1016/j.appet.2018.05.142

[24] University of Sydney the official website of the glycemic index and GI database. https://glycemicindex.com/

[25] Atkinson FS, Foster-Powell K, Brand-Miller JC (2008) International tables of glycemic index and glycemic load values: 2008. Diabetes Care 31:2281–2283. 10.2337/dc08-1239 18835944 10.2337/dc08-1239 PMC2584181

[26] Buyken AE, Dettmann W, Kersting M et al (2005) Glycaemic index and glycaemic load in the diet of healthy schoolchildren: trends from 1990 to 2002, contribution of different carbohydrate sources and relationships to dietary quality. Br J Nutr 94:796–803. 10.1079/BJN20051537 16277784 10.1079/bjn20051537

[27] Herder C, Bongaerts BWC, Rathmann W et al (2013) Association of subclinical inflammation with polyneuropathy in the older population: KORA F4 study. Diabetes Care 36:3663–3670. 10.2337/dc13-0382 24009302 10.2337/dc13-0382 PMC3816905

[28] Goletzke J, Buyken AE, Joslowski G et al (2014) Increased intake of carbohydrates from sources with a higher glycemic index and lower consumption of whole grains during puberty are prospectively associated with higher IL-6 concentrations in younger adulthood among healthy individuals. J Nutr 144:1586–1593. 10.3945/jn.114.193391 25080538 10.3945/jn.114.193391

[29] Hatziagelaki E, Herder C, Tsiavou A et al (2015) Serum chemerin concentrations associate with beta-cell function, but not with insulin resistance in individuals with non-alcoholic fatty liver disease (NAFLD). PLoS ONE 10:e0124935. 10.1371/journal.pone.0124935 25933030 10.1371/journal.pone.0124935 PMC4416815

[30] Herder C, Ouwens DM, Carstensen M et al (2015) Adiponectin may mediate the association between omentin, circulating lipids and insulin sensitivity: results from the KORA F4 study. Eur J Endocrinol 172:423–432. 10.1530/EJE-14-0879 25733068 10.1530/EJE-14-0879

[31] Radcliffe Department of Medicine HOMA Calculator. https://www.rdm.ox.ac.uk/about/our-clinical-facilities-and-units/DTU/software/homa. Accessed 01 Dec 2024

[32] Wallace TM, Levy JC, Matthews DR (2004) Use and abuse of HOMA modeling. Diabetes Care 27:1487–1495. 10.2337/diacare.27.6.1487 15161807 10.2337/diacare.27.6.1487

[33] Lee J-H, Kim D, Kim HJ et al (2010) Hepatic steatosis index: a simple screening tool reflecting nonalcoholic fatty liver disease. Dig Liver Dis 42:503–508. 10.1016/j.dld.2009.08.002 19766548 10.1016/j.dld.2009.08.002

[34] Bedogni G, Bellentani S, Miglioli L et al (2006) The fatty liver index: a simple and accurate predictor of hepatic steatosis in the general population. BMC Gastroenterol 6:33. 10.1186/1471-230X-6-33 17081293 10.1186/1471-230X-6-33 PMC1636651

[35] Hohoff E, Jankovic N, Perrar I et al (2024) The association between dairy intake in adolescents on inflammation and risk markers of type 2 diabetes during young adulthood: results of the DONALD study. Public Health Nutr 27:e91. 10.1017/S1368980024000624 38477143 10.1017/S1368980024000624 PMC10966841

[36] Roenneberg T, Wirz-Justice A, Merrow M (2003) Life between clocks: daily temporal patterns of human chronotypes. J Biol Rhythms 18:80–90. 10.1177/0748730402239679 12568247 10.1177/0748730402239679

[37] Roenneberg T, Pilz LK, Zerbini G et al (2019) Chronotype and social jetlag: a (self-) critical review. Biology (Basel) 8:54. 10.3390/biology8030054 31336976 10.3390/biology8030054 PMC6784249

[38] Roenneberg T, Kuehnle T, Pramstaller PP et al (2004) A marker for the end of adolescence. Curr Biol 14:R1038–R1039. 10.1016/j.cub.2004.11.039 15620633 10.1016/j.cub.2004.11.039

[39] Robert-Koch-Institut (2013) Beiträge zur Gesundheitsberichterstattung des Bundes: Referenzperzentile für anthropometrische Maßzahlen und Blutdruck aus der Studie zur Gesundheit von Kindern und Jugendlichen in Deutschland (KiGGS): 2. erweiterte Auflage

[40] Schofield WN (1985) Predicting basal metabolic rate, new standards and review of previous work. Hum Nutr Clin Nutr 39(Suppl 1):5–41 4044297

[41] Ainsworth BE, Haskell WL, Herrmann SD, Meckes N, Bassett DR, Tudor-Locke C, Leon AS (2011) Compendium of physical activities: a second update of codes and MET values. Med Sci Sports Exercise 43(8):1575–1581 10.1249/MSS.0b013e31821ece12 21681120

[42] Knol MJ, VanderWeele TJ (2012) Recommendations for presenting analyses of effect modification and interaction. Int J Epidemiol 41:514–520. 10.1093/ije/dyr218 22253321 10.1093/ije/dyr218 PMC3324457

[43] Willett WC, Howe GR, Kushi LH (1997) Adjustment for total energy intake in epidemiologic studies. Am J Clin Nutr 65:1220S-1228S; discussion 1229S-1231S. 10.1093/ajcn/65.4.1220S 10.1093/ajcn/65.4.1220S 9094926

[44] Buyken AE, Goletzke J, Joslowski G et al (2014) Association between carbohydrate quality and inflammatory markers: systematic review of observational and interventional studies. Am J Clin Nutr 99:813–833. 10.3945/ajcn.113.074252 24552752 10.3945/ajcn.113.074252

[45] Textor J, Hardt J, Knüppel S (2011) DAGitty: a graphical tool for analyzing causal diagrams. Epidemiology 22 10.1097/EDE.0b013e318225c2be 21811114

[46] Sichert-Hellert W, Kersting M, Schöch G (1998) Underreporting of energy intake in 1 to 18 year old German children and adolescents. Eur J Nutr 37:242–251. 10.1007/s003940050023 10.1007/s003940050023 9800315

[47] McHill AW, Czeisler CA, Phillips AJK et al (2019) Caloric and macronutrient intake differ with circadian phase and between lean and overweight young adults. Nutrients. 10.3390/nu11030587 30862011 10.3390/nu11030587 PMC6471585

[48] Xiao Q, Garaulet M, Scheer FAJL (2019) Meal timing and obesity: interactions with macronutrient intake and chronotype. Int J Obes (Lond) 43:1701–1711. 10.1038/s41366-018-0284-x 30705391 10.1038/s41366-018-0284-x PMC6669101

[49] Mazri FH, Manaf ZA, Shahar S et al (2021) Do temporal eating patterns differ in healthy versus unhealthy overweight/obese individuals? Nutrients. 10.3390/nu13114121 34836375 10.3390/nu13114121 PMC8618797

[50] Kalsbeek A, La Fleur S, Fliers E (2014) Circadian control of glucose metabolism. Mol Metab 3:372–383. 10.1016/j.molmet.2014.03.002 24944897 10.1016/j.molmet.2014.03.002 PMC4060304

[51] Morris CJ, Yang JN, Garcia JI et al (2015) Endogenous circadian system and circadian misalignment impact glucose tolerance via separate mechanisms in humans. Proc Natl Acad Sci U S A 112:E2225–E2234. 10.1073/pnas.1418955112 25870289 10.1073/pnas.1418955112 PMC4418873

[52] Neuenschwander M, Ballon A, Weber KS et al (2019) Role of diet in type 2 diabetes incidence: umbrella review of meta-analyses of prospective observational studies. BMJ 366:l2368. 10.1136/bmj.l2368 31270064 10.1136/bmj.l2368 PMC6607211

[53] Miller V, Jenkins DA, Dehghan M et al (2024) Associations of the glycaemic index and the glycaemic load with risk of type 2 diabetes in 127 594 people from 20 countries (PURE): a prospective cohort study. Lancet Diabetes Endocrinol 12:330–338. 10.1016/S2213-8587(24)00069-X 38588684 10.1016/S2213-8587(24)00069-X

[54] Buyken AE, Trauner K, Günther ALB et al (2007) Breakfast glycemic index affects subsequent daily energy intake in free-living healthy children. Am J Clin Nutr 86:980–987. 10.1093/ajcn/86.4.980 17921374 10.1093/ajcn/86.4.980

[55] Ahola AJ, Mutter S, Forsblom C et al (2019) Meal timing, meal frequency, and breakfast skipping in adult individuals with type 1 diabetes - associations with glycaemic control. Sci Rep 9:20063. 10.1038/s41598-019-56541-5 31882789 10.1038/s41598-019-56541-5 PMC6934661

[56] Ruddick-Collins LC, Morgan PJ, Fyfe CL et al (2022) Timing of daily calorie loading affects appetite and hunger responses without changes in energy metabolism in healthy subjects with obesity. Cell Metab 34:1472-1485.e6. 10.1016/j.cmet.2022.08.001 36087576 10.1016/j.cmet.2022.08.001 PMC9605877

[57] Bonsembiante L, Targher G, Maffeis C (2021) Type 2 diabetes and dietary carbohydrate intake of adolescents and young adults: what is the impact of different choices? Nutrients. 10.3390/nu13103344 34684345 10.3390/nu13103344 PMC8537173

[58] Alexy U, Wicher M, Kersting M (2010) Breakfast trends in children and adolescents: frequency and quality. Public Health Nutr 13:1795–1802. 10.1017/S1368980010000091 20236559 10.1017/S1368980010000091

[59] Mantovani A, Byrne CD, Bonora E et al (2018) Nonalcoholic fatty liver disease and risk of incident type 2 diabetes: a meta-analysis. Diabetes Care 41:372–382. 10.2337/dc17-1902 29358469 10.2337/dc17-1902

[60] Goletzke J, Herder C, Joslowski G et al (2013) Habitually higher dietary glycemic index during puberty is prospectively related to increased risk markers of type 2 diabetes in younger adulthood. Diabetes Care 36:1870–1876. 10.2337/dc12-2063 23349549 10.2337/dc12-2063 PMC3687320

[61] Valdés J, Rodríguez-Artalejo F, Aguilar L et al (2013) Frequency of family meals and childhood overweight: a systematic review. Pediatr Obes 8:e1–e13. 10.1111/j.2047-6310.2012.00104.x 23239547 10.1111/j.2047-6310.2012.00104.x

[62] Bokhof B, Günther ALB, Berg-Beckhoff G et al (2010) Validation of protein intake assessed from weighed dietary records against protein estimated from 24 h urine samples in children, adolescents and young adults participating in the Dortmund Nutritional and Longitudinally Designed (DONALD) Study. Public Health Nutr 13:826–834. 10.1017/S136898000999317X 20074394 10.1017/S136898000999317X

[63] Zavada A, Gordijn MCM, Beersma DGM et al (2005) Comparison of the Munich chronotype questionnaire with the Horne-Ostberg’s morningness-eveningness score. Chronobiol Int 22:267–278. 10.1081/cbi-200053536 16021843 10.1081/cbi-200053536

[64] Roenneberg T, Merrow M (2007) Entrainment of the human circadian clock. Cold Spring Harb Symp Quant Biol 72:293–299. 10.1101/sqb.2007.72.043 18419286 10.1101/sqb.2007.72.043

[65] Rothman KJ (2002) Epidemiology: an introduction. Oxford University Press, New York, NY

[66] Kalmbach DA, Schneider LD, Cheung J et al (2017) Genetic basis of chronotype in humans: insights from three landmark GWAS. Sleep. 10.1093/sleep/zsw048 28364486 10.1093/sleep/zsw048 PMC6084759

