# Supplementary material for: Circadian distribution of carbohydrate intake during adolescence and association with risk markers of type 2 diabetes in adulthood—the role of chronotype
Source: Eur J Nutr. 2026 Aug 3;65(5):228. doi: 10.1007/s00394-026-04056-x (PMC13433581; doi:10.1007/s00394-026-04056-x)
Supplement: Supplementary file 1 [file 394_2026_4056_MOESM1_ESM.docx]

**Supplementary Files**

**Figure S1: Study Flow Diagram for participant data from the DONALD Study**

^1^According to Perrar et al. 2023.

^2^Blood measures start at age ≥18 years onwards.

^3^MCTQ assessment starts at age 9 years onwards.

^4^Adolescence was defined in accordance with our previous publications as follows. Girls: 9 - 15 years and boys: 10 – 16 years.

^5^Replaced by an alternative measure from the source of standard analytes.

**Figure S2: DAG accompanying the association between circadian intake of carbohydrates and quality and diabetes risk.**

**Derived minimally sufficient adjustment sets (MSAS)^1^:**

- BMI-SDS at baseline, early life factors, age at outcome assessment, birth weight, chronotype, fat, history of diabetes (parents), physical activity, protein intake, sex, smoking in the household, social jetlag, total energy intake
- BMI-SDS at baseline, early life factors, birth weight, chronotype, fat, history of diabetes (parents), physical activity, protein intake, sex, smoking in the household, social jetlag, time between measurements, total energy intake
- BMI-SDS at baseline, age at diet intake measurement, chronotype, fat, protein intake, sex, social jetlag, total energy intake
- BMI-SDS at baseline, age at outcome assessment, birth weight, chronotype, fat, history of diabetes (parents), maternal education/occupation, physical activity, protein intake, sex, smoking in the household, social jetlag, total energy intake^2^
- BMI-SDS at baseline, birth weight, chronotype, fat, history of diabetes (parents), maternal education/occupation, physical activity, protein intake, sex, smoking in the household, social jetlag, time between measurements, total energy intake

^1^According to Textor et al. 2016

^2^Chosen set of covariates, variables were further selected to reduce multicollinearity (e.g. either chronotype or SJL), to omit substitution modelling (e.g. fat and protein intake) or to fulfil the requirements of the sample under study (e.g. history of diabetes which was excluded prior analyses). The final model included the following variables (e.g. Table 3 and Figure 1): BMI-SDS, age at blood withdrawl and date of dietary record (improved precision of the estimate), birthweight, parental employment, education, MET-min, residuals of animal protein, sex, smoking (yes/no), social jetlag, total kcal intake.

^3^ Pubertal status, screen time and sleep duration were also included in the DAG but did not emerge in any of the MSAS.

Source Code Dagitty:

dag {"BMI-SDS adult" [pos="-0.552,-1.265"]"BMI-SDS at baseline" [pos="-1.325,-0.840"]"CHO intake morning/evening" [exposure,pos="-1.540,0.419"]"early life factors" [pos="-0.892,-0.245"]"age at diet intake measurement" [pos="-1.710,-0.560"]"age at outcome assessment" [pos="-1.053,-1.223"]"birth weight" [pos="-0.351,-0.731"]"history of diabetes (parents)" [pos="-0.876,0.899"]"inflammation and diabetes" [outcome,pos="-0.188,0.367"]"maternal education/occupation" [pos="-0.265,-0.340"]"physical activity" [pos="-1.845,1.201"]"protein intake" [pos="-1.481,1.331"]"smoking in the household" [pos="-0.313,1.370"]"social jetlag" [pos="-0.721,0.618"]"time between measurements" [pos="-1.263,-0.219"]"total energy intake" [pos="-1.103,0.704"]chronotype [pos="-0.911,0.261"]fat [pos="-1.039,1.353"]sex [pos="-1.895,0.100"]"BMI-SDS at baseline" -> "BMI-SDS adult""BMI-SDS at baseline" -> "CHO intake morning/evening""BMI-SDS at baseline" -> "inflammation and diabetes""CHO intake morning/evening" -> "BMI-SDS adult""CHO intake morning/evening" -> "inflammation and diabetes""CHO intake morning/evening" <-> "protein intake""CHO intake morning/evening" <-> fat"early life factors" -> "inflammation and diabetes""age at diet intake measurement" -> "BMI-SDS at baseline""age at diet intake measurement" -> "CHO intake morning/evening""age at outcome assessment" -> "BMI-SDS adult""age at outcome assessment" -> "inflammation and diabetes""birth weight" -> "BMI-SDS adult""birth weight" -> "BMI-SDS at baseline""birth weight" -> "inflammation and diabetes""history of diabetes (parents)" -> "BMI-SDS adult""history of diabetes (parents)" -> "BMI-SDS at baseline""history of diabetes (parents)" -> "birth weight""history of diabetes (parents)" -> "inflammation and diabetes""maternal education/occupation" -> "BMI-SDS adult""maternal education/occupation" -> "BMI-SDS at baseline""maternal education/occupation" -> "early life factors""maternal education/occupation" -> "physical activity""maternal education/occupation" -> "smoking in the household""physical activity" -> "BMI-SDS adult""physical activity" -> "BMI-SDS at baseline""physical activity" -> "inflammation and diabetes""protein intake" -> "inflammation and diabetes""protein intake" <-> fat"smoking in the household" -> "BMI-SDS adult""smoking in the household" -> "BMI-SDS at Baseline""smoking in the household" -> "early life factors""smoking in the household" -> "birth weight""smoking in the household" -> "inflammation and diabetes""social jetlag" -> "CHO intake morning/evening""social jetlag" -> "inflammation and diabetes""time between measurements" -> "age at diet intake measurement""time between measurements" -> "age at outcome assessment""total energy intake" -> "CHO intake morning/evening""total energy intake" -> "inflammation and diabetes"chronotype -> "BMI-SDS adult"chronotype -> "CHO intake morning/evening"chronotype -> "inflammation and diabetes"chronotype -> "social jetlag"chronotype -> "total energy intake"fat -> "inflammation and diabetes"sex -> "BMI-SDS adult"sex -> "BMI-SDS at Baseline"sex -> "CHO intake morning/evening"sex -> "inflammation and diabetes"sex -> "protein intake"sex -> "total energy intake"sex -> chronotype}

**Table S1: Regression coefficients and 95% confidence intervals between morning (before 11 a.m.) or evening (after 6 p.m.) carbohydrate intake or quality during adolescence and HOMA2-IR in adulthood stratified by chronotype (N = 224), the DONALD study.**

Table S1 to be continued…

Table S1 continued:

**Table S2: Regression coefficients and 95% CI of the association between “Eveningness in CHO intake”^a^ and HOMA2-IR, (N (percent boys/girls) = 224 (44 / 56))**

|  | **HOMA2 IR** | | |  |  |
| --- | --- | --- | --- | --- | --- |
|  | **T1 More**  **Morningness according to the “Eveningness score”** | **T2 Moderate**  **Morningness**  **according to the “Eveningness score”** | **T3 More**  **Eveningness according to the “Eveningness score”** | **P_t_*_rend_^b^*** | **P*_interaction_*** |
| **Early chronotype** | | | |  |  |
| **N (percent boys/girls)** | 47 (36/64) | 32 (47/53) | 31 (48/52) |  |  |
| **Eveningness score** | -11.67 (-14.32; -9.64) | -6.17 (-7.53; -3.21) | 1.20 (-0.91; 4.04) |  |  |
| **Crude**^c^ | 1.00 | 0.06 (-0.05; 0.17) | 0.13 (0.03; 0.17) |  |  |
| **Adjusted**^d^ | 1.00 | 0.06 (-0.05; 0.17) | 0.12 (0.01; 0.23) | 0.03 |  |
| **lsmeans HOMA2-IR** | 1.73 (1.16; 2.40) | 2.22 (1.66: 2.87) | 2.79 (2.20; 3.46) |  |  |
|  |  |  |  |  |  |
| **Late chronotype** | | | |  |  |
| **N (percent boys/girls)** | 26 (54/46) | 43 (40/60) | 45 (38/62) |  |  |
| **Eveningness score** | -13.32 (-16.17; -11.92) | -6.31 (-7.93; -4.16) | 2.85 (-0.45; 6.87) |  |  |
| **Crude**^c^ | 1.00 | -0.14 (-0.25; -0.03) | -0.04 (-0.15; 0.07) |  |  |
| **Adjusted**^d^ | 1.00 | -0.15 (-0.25; -0.05) | -0.02 (-0.13; 0.09) | 0.60 |  |
| **lsmeans HOMA2-IR** | 3.12 (2.49; 3.82) | 1.84 (1.33: 2.44) | 2.95 (2.23; 3.79) |  |  |
|  |  |  |  |  | *0.01* |
|  | | | |  |  |
| **Eveningness CHO low GI** | -11.30 (-15.07; -9.11) | -2.85 (-4.22; -1.67) | 5.24 (2.90; 9.26) |  |  |
| **Crude^c^** | 1.00 | 0.04 (-0.04; 0.11) | -0.01 (-0.09; 0.06) |  |  |
| **Adjusted**^d^ | 1.00 | 0.04 (-0.04; 0.11) | 0.00 (-0.07; 0.08) | 0.75 |  |
| **lsmeans HOMA2 IR** | 2.37 (1.95; 2.85) | 2.80 (2.32; 3.32) | 2.24 (1.83; 2.70) |  |  |
|  |  |  |  |  | *0.18* |
| **Eveningness CHO higher GI** | -10.00 (-14.47; -8.18) | -2.56 (-3.98; -0.82) | 5.64 (3.47; 9.23) |  |  |
| **Crude**^c^ | 1.00 | 0.00 (-0.07; 0.08) | -0.01 (-0.08; 0.07) |  |  |
| **Adjusted**^d^ | 1.00 | 0.02 (-0.06; 0.09) | 0.03 (-0.05; 0.11) | 0.45 |  |
| **lsmeans HOMA2 IR** | 2.31 (1.87; 2.80) | 2.46 (2.01: 2.95) | 2.62 (2.16; 3.12) |  |  |
|  |  |  |  |  | *0.13* |

^a^The circadian distribution of diurnal CHO intake was measured by 'Eveningness in CHO intake' (E%) and calculated as: CHOevening-CHOmorning.

^b^P_trend_ were derived by using the median of the categorical exposure variables in the model.

^c^Crude models included sex, age at blood withdrawal.

^d^The adjusted model was additionally adjusted for: SDS-BMI, residual of animal protein intake, total kcal/d, smoking (yes/no), MET-min, parental employment, parental education, birthweight.

**Table S3: Regression coefficients and 95% CI of the association between “Eveningness in CHO intake”^a^ and hepatic steatosis index (HSI), (N (percent boys/girls) = 227 (42/ 58)).**

|  | **HSI** | | |  |  |
| --- | --- | --- | --- | --- | --- |
|  | **T1 More**  **Morningness according to the “Eveningness score”** | **T2 Moderate**  **Morningness**  **according to the “Eveningness score”** | **T3 More**  **Eveningness according to the “Eveningness score”** | **P_t_*_rend_^b^*** | **P_interaction_** |
| **N (percent boys/girls)** | 75 (43/57) | 76 (42/58) | 76 (42/58) |  |  |
| **Eveningness score CHO** | -13.04 (-15.41; -10.38) | -6.21 (-7.66; -3.85) | 2.38 (-0.81; 4.94) |  |  |
| **Crude**^c^ | 1.00 | -0.51 (-2.29; 1.26) | 0.31 (-1.47; 2.08) | *0.69* |  |
| **Adjusted**^d^ | 1.00 | -0.71 (-2.27; 0.84) | 0.06 (-1.54; 1.67) | *0.90* |  |
| **lsmeans HSI** | 32.53 (31.28; 33.79) | 33.35 (32.10; 34.61) | 33.05 (31.79; 34.31) |  |  |
|  |  |  |  |  | *0.15* |
| **Eveningness CHO low GI** | -11.30 (-15.07; -9.11) | -2.85 (-4.22; -1.67) | 5.24 (2.90; 9.26) |  |  |
| **Crude**^c^ | 1.00 | 1.17 (-0.60; 2.94) | 0.96 (-0.82; 2.73) | *0.29* |  |
| **Adjusted**^d^ | 1.00 | 1.42 (-0.15; 2.98) | 0.30 (-1.28; 1.87) | *0.52* |  |
| **lsmeans HSI** | 32.70 (30.96; 34.45) | 33.09 (31.56; 34.62) | 33.55 (32.15; 34.96) |  |  |
|  |  |  |  |  | *0.59* |
| **Eveningness CHO higher GI** | -10.00 (-14.47; -8.18) | -2.56 (-3.98; -0.82) | 5.64 (3.47; 9.23) |  |  |
| **Crude**^c^ | 1.00 | -0.39 (-2.17; 1.38) | -1.03 (-2.80; 0.74) | *0.25* |  |
| **Adjusted**^d^ | 1.00 | 0.08 (-1.53; 1.70) | 0.08 (-1.55; 1.72) | *0.77* |  |
| **lsmeans HSI** | 32.92 (31.78; 34.06) | 33.00 (31.89; 34.11) | 33.00 (31.88; 34.11) |  |  |
|  |  |  |  |  | *0.60* |

^a^The circadian distribution of diurnal CHO intake was measured by 'Eveningness in CHO intake' (E%) and calculated as: CHOevening-CHOmorning.

^b^P_trend_ were derived by using the median of the categorical exposure variables in the model.

^c^Crude models included sex, age at blood withdrawal.

^d^The adjusted model was additionally adjusted for: SDS-BMI, residual of animal protein intake, total kcal/d, smoking (yes/no), MET-min, parental employment, parental education, birth weight.

**Table S4: Regression coefficients and 95% CI of the association between “Eveningness in CHO intake”^a^ and fatty liver index (FLI), (N total (percent boys/girls) = (227(42/48)).**

|  | **FLI** | | |  |  |
| --- | --- | --- | --- | --- | --- |
|  | **T1 More**  **Morningness according to the “Eveningness score”** | **T2 Moderate**  **Morningness**  **according to the “Eveningness score”** | **T3 More**  **Eveningness according to the “Eveningness score”** | **P_t_*_rend_^b^*** | **P*_interaction_*** |
|  |  |  |  |  |  |
| **N (percent boys/girls)** | 75 (43 / 57) | 76 (42 / 58) | 76 ( 42 / 58) |  |  |
| **Eveningness score CHO** | -13.04 (-15.41; -10.38) | -6.21 (-7.66; -3.85) | 2.37 (-0.81; 4.94) |  |  |
| **Crude**^c^ | 1.00 | 1.07 (-5.51; 7.67) | 2.29 (-4.30; 8.89) | 0.49 |  |
| **Adjusted**^d^ | 1.00 | 0.79 (-5.20; 6.78) | 2.44 (-3.76; 8.65) | 0.43 |  |
| **lsmeans FLI** | 16.04 (11.76; 20.32) | 16.49 (12.29; 20.70) | 18.04 (13.74; 22.34) |  |  |
|  |  |  |  |  | *0.10* |
| **Eveningness CHO low GI** |  |  |  |  |  |
| **Crude**^c^ | 1.00 | 0.06 (-0.07; 0.18) | 0.03 (-0.10; 0.15) | 0.76 |  |
| **Adjusted**^d^ | 1.00 | 0.07 (-0.04; 0.18) | -0.01 (-0.12; 0.10) | 0.80 |  |
| **lsmeans FLI** | 16.18 (11.94; 20.41) | 19.12 (14.88; 23.35) | 15.32 (11.09; 19.56) |  |  |
|  |  |  |  |  | *0.19* |
| **Eveningness CHO higher GI** | -10.00 (-14.47; -8.18) | -2.56 (-3.98; -0.82) | 5.64 (3.47; 9.23) |  |  |
| **Crude**^c^ | 1.00 | -2.96 (-9.56; 3.63) | -1.55 (-8.13; 5.03) | 0.66 |  |
| **Adjusted**^d^ | 1.00 | -0.90 (-7.07; 5.27) | 2.71 (-3.53; 8.94) | 0.37 |  |
| **lsmeans FLI** | 16.25 (11.90; 20.60 | 15.35 (11.11; 19.60) | 18.96 (14.70; 23.22) |  |  |
|  |  |  |  |  | *0.58* |

^a^The circadian distribution of diurnal CHO intake was measured by 'Eveningness in CHO intake' (E%) and calculated as: CHOevening-CHOmorning.

^b^P_trend_ were derived by using the median of the categorical exposure variables in the model.

^c^Crude models included sex, age at blood withdrawal.

^d^The adjusted model was additionally adjusted for: SDS-BMI, residual of animal protein intake, total kcal/d, smoking (yes/no), MET-min, parental employment, parental education, birthweight.

**Table S5: Regression coefficients and 95% CI of the association between “Eveningness in CHO intake”^a^ and the pro-inflammatory score (PIS), (N total (percent boys/girls) = 227(42.33/47.67)).**

|  | **Pro-inflammatory score** | | |  |  |
| --- | --- | --- | --- | --- | --- |
|  | **T1 More**  **Morningness according to the “Eveningness score”** | **T2 Moderate**  **Morningness**  **according to the “Eveningness score”** | **T3 More**  **Eveningness according to the “Eveningness score”** | **P_t_*_rend_^b^*** | **P*_interaction_*** |
| **Eveningness** | -5.78 (-9.93; -3.61) | 2.67 (-0.06; 3.85) | 11.56 (8.78; 15.28) |  |  |
| **N (percent boys/girls)** | 75 (43/57) | 76 (42/58) | 76 (42/58) |  |  |
| **Eveningness score CHO** | -13.04 (-15.41; -10.38) | -6.21 (-7.66; -3.85) | 2.38 (-0.81; 4.94) |  |  |
| **Crude**^c^ | 1.00 | -0.03 (-0.09; 0.03) | -0.02 (-0.07; 0.04) | 0.64 |  |
| **Adjusted**^d^ | 1.00 | -0.03 (-0.09; 0.03) | -0.01 (-0.07; 0.05) | 0.73 |  |
| **lsmeans PIS** | 1.20 (1.11; 1.29) | 1.13 (1.04; 1.21) | 1.17 (1.08; 1.26) |  |  |
|  |  |  |  |  | *0.83* |
| **Eveningness CHO low GI** | -11.30 (-15.07; -9.11) | -2.85 (-4.22; -1.67) | 5.24 (2.90; 9.26) |  |  |
| **Crude**^c^ | 1.00 | 0.04 (-0.14; 0.22) | 0.02 (-0.16; 0.21) | 0.81 |  |
| **Adjusted**^d^ | 1.00 | 0.04 (-0.14; 0.22) | 0.01 (-0.19; 0.18) | 0.96 |  |
| **lsmeans PIS** | 1.15 (1.07; 1.25) | 1.17 (1.09; 1.27) | 1.16 (1.08; 1.25) |  |  |
|  |  |  |  |  | *0.76* |
| **Eveningness CHO higher GI** | -10.00 (-14.47; -8.18) | -2.56 (-3.98; -0.82) | 5.64 (3.47; 9.23) |  |  |
| **Crude**^c^ | 1.00 | -0.01 (-0.07; 0.05) | -0.04 (-0.10; 0.02) | 0.20 |  |
| **Adjusted**^d^ | 1.00 | 0.00 (-0.06; 0.06) | -0.01 (-0.08; 0.05) | 0.62 |  |
| **lsmeans PIS** | 1.18 (1.09; 1.27) | 1.18 (1.09; 1.27) | 1.14 (1.06; 1.23) |  |  |
|  |  |  |  |  | *0.54* |

^a^The circadian distribution of diurnal CHO intake was measured by 'Eveningness in CHO intake' (E%) and calculated as: CHOevening-CHOmorning.

^b^P_trend_ were derived by using the median of the categorical exposure variables in the model.

^c^Crude models included sex, age at blood withdrawal.

^d^The adjusted model was additionally adjusted for: SDS-BMI, residual of animal protein intake, total kcal/d, smoking (yes/no), MET-min, parental employment, parental education, birth weight.

Literature accompanying the appendix

Perrar, I., Alexy, U. & Nöthlings, U. Cohort profile update–overview of over 35 years of research in the Dortmund Nutritional and Anthropometric Longitudinally Designed (DONALD) study. *Eur J Nutr* **63**, 727–740 (2024). <https://doi.org/10.1007/s00394-023-03290-x>

Johannes Textor, Benito van der Zander, Mark K. Gilthorpe, Maciej Liskiewicz, George T.H. Ellison.
[Robust causal inference using directed acyclic graphs: the R package 'dagitty'.](http://dx.doi.org/10.1093/ije/dyw341)
*International Journal of Epidemiology* 45(6):1887-1894 (2016)
